# Omics in a Digital World: The Role of Bioinformatics in Providing New Insights Into Human Aging

**DOI:** 10.3389/fgene.2021.689824

**Published:** 2021-06-10

**Authors:** Serena Dato, Paolina Crocco, Nicola Rambaldi Migliore, Francesco Lescai

**Affiliations:** ^1^Department of Biology, Ecology and Earth Sciences, University of Calabria, Rende, Italy; ^2^Department of Biology and Biotechnology “L. Spallanzani”, University of Pavia, Pavia, Italy

**Keywords:** bioinformatics, systems biology, aging, translational genomics, regulation, proteomics, metabolomics, databases

## Abstract

**Background:**

Aging is a complex phenotype influenced by a combination of genetic and environmental factors. Although many studies addressed its cellular and physiological age-related changes, the molecular causes of aging remain undetermined. Considering the biological complexity and heterogeneity of the aging process, it is now clear that full understanding of mechanisms underlying aging can only be achieved through the integration of different data types and sources, and with new computational methods capable to achieve such integration.

**Recent Advances:**

In this review, we show that an omics vision of the age-dependent changes occurring as the individual ages can provide researchers with new opportunities to understand the mechanisms of aging. Combining results from single-cell analysis with systems biology tools would allow building interaction networks and investigate how these networks are perturbed during aging and disease. The development of high-throughput technologies such as next-generation sequencing, proteomics, metabolomics, able to investigate different biological markers and to monitor them simultaneously during the aging process with high accuracy and specificity, represents a unique opportunity offered to biogerontologists today.

**Critical Issues:**

Although the capacity to produce big data drastically increased over the years, integration, interpretation and sharing of high-throughput data remain major challenges. In this paper we present a survey of the emerging omics approaches in aging research and provide a large collection of datasets and databases as a useful resource for the scientific community to identify causes of aging. We discuss their peculiarities, emphasizing the need for the development of methods focused on the integration of different data types.

**Future Directions:**

We critically review the contribution of bioinformatics into the omics of aging research, and we propose a few recommendations to boost collaborations and produce new insights. We believe that significant advancements can be achieved by following major developments in bioinformatics, investing in diversity, data sharing and community-driven portable bioinformatics methods. We also argue in favor of more engagement and participation, and we highlight the benefits of new collaborations along these lines. This review aims at being a useful resource for many researchers in the field, and a call for new partnerships in aging research.

## Introduction

Over the past decades, the average human life expectancy has increased dramatically, by more than 2 years per decade (Oeppen and Vaupel, 2002; Vaupel, 2010; Meyer et al., 2020). In 2021, the worldwide life expectancy is estimated to be 72.81 years, a 0.24% increase from 2020 (Parant, 1990), although the prevision does not take into account the impact of the COVID-19 virus on mortality (Goldstein and Lee, 2020; Kontis et al., 2020). This increase in life expectancy, however, does not parallel with an equivalent increase in disease-free lifespan or healthspan: this is why biogerontologists are nowadays struggling with identifying actionable mechanisms of aging, with the goal of extending the time individual lives in good health, possibly delaying age-related diseases, and therefore reaching longevity. The issue is not simple to solve. In fact, although our understanding of aging biology in model systems has increased dramatically, thanks to the possibility to model the effect of single variants on the probability to extend our lifespan, Human aging and longevity are complex polygenic traits. They are influenced by the inheritance pattern of multiple genes/variants, each one with pleiotropic protective roles across several age-related diseases, and their interaction with environment. People can achieve older age while suffering major age-related diseases, because of their capability to survive those disorders, or they can escape entirely some of the most frequent causes of death and impairment, thus living not just a long but also a healthy life. The difference between these two aging trajectories and phenotypes is greatly discussed and investigated: many studies aimed at determining the relative contribution of the many players involved in this challenge, which include diet, gender, education, socioeconomic status, social engagement, access to medical care and, last but not least, genetics. For these reasons, many disciplines are involved in the search for contributors to human aging, from biology to medicine, bioinformatics, demography, sociology, psychology, and economy. This large effort in recent years led to an integrated view of aging, in which health and diseases can be considered part of a continuum (Franceschi et al., 2018) where boundaries do not exist and the two extremes are represented by centenarians, who largely avoided or postponed most diseases and experience a decelerated aging, and patients who suffered one or more severe diseases starting from their 60 s, 70 s, or 80 s and show signs of accelerated aging. As part of a continuum, health and diseases share the same underpinning mechanisms. Thus, it is likely that risk alleles exerting its effect on the susceptibility to common age-related diseases influence the individual lifespan, and the difference in clinical manifestations is the result of peculiar combinations of alterations affecting the same, limited set of basic pathways shared with the aging process. This hypothesis is the leading argument, which guided the efforts to identify genetic variation associated with human aging. Large studies confirmed this integrated view. As an example, in 2014, a meta-analysis on the genetics of human longevity (Deelen et al., 2014) identified an intergenic region on chromosome 5q33.3 promoting human longevity and associated with lower mortality risk for CVD, decreased risk for coronary artery disease, lower diastolic and systolic blood pressure. On the other hand, studies investigating families of long-lived individuals (LLI) not only demonstrated that first degree relatives of centenarians show greater chances of living to old ages too, as compared to the general population (Sebastiani et al., 2016), but they are also more likely to show delayed onset of age-related diseases and compressed disease morbidity (Gudmundsson et al., 2000; Terry et al., 2003; Atzmon et al., 2004; Lipton et al., 2010; Dutta et al., 2013).

Among the cellular mechanisms influencing health and shared with common diseases, experts suggest seven master regulators, represented by adaptation to stress, loss of proteostasis, stem cell exhaustion, metabolic derangement, macromolecular damage, epigenetic modifications, and inflammation (Kennedy et al., 2014; Franceschi et al., 2018).

## Overview: Cohort Aging Studies Collecting Omics Data

The search for determinants of aging is nowadays involving the collection of large cohorts of individuals to perform longitudinal studies. More than 70 community-based cohort studies have been conducted, mostly in North America or Northern Europe, 51 approved by NIA (National Institute of Ageing) (see https://www.ncbi.nlm.nih.gov/pmc/articles/PMC3135270/ for a complete list), either including exclusively elderly people or following people from middle-age (50+ years at enrollment) to death, with a mean follow-up period of 10 years. Usually, collected variables comprise data on familiar composition, employment, economic status (socio-demographical variables), self-reported chronic diseases and determination of the functional status, through anthropometrics measures and test of physical performances, measures of cognition, and, for about 60% of the studies, the collection of biological samples. One third of all the studies also conducted genetic analyses (Seematter-Bagnoud and Santos-Eggimann, 2006). A review of major cohorts and longitudinal studies still ongoing, can be found in Stanziano et al. (2010) and we report some of the more relevant cohort studies in Table 1.

**TABLE 1:** Relevant cohort studies in aging research.

Study/Cohort	Aim of the research	Main publication	Population (N samples*)	Start date Status: Completed/ongoing
The Long Life Family Study	Family based, longitudinal study of healthy aging and longevity launched in 2005 aimed at the identification of markers in blood able to predict survival, better physical function, disease-free aging, dementia, and cardiovascular disease.	Sebastiani et al., 2017	Caucasian and American of European ancestry (535 families, 1.499 individuals in the Proband Generation, 2.594 individuals in the Offspring Generation and 830 Spouse Controls)	Started in 2005 Status: Completed (2009) https://www.sdu.dk/en/llfs
The Healthy Ageing in Neighborhoods of Diversity across the Life Span (HANDLS) study	Prospective study, aimed at investigating the influence of age, race, socioeconomic status on health and on major age-related diseases or conditions (Diabetes, Cerebrovascular Disease, Age-Associated Decline, Cardiovascular Disease)	Evans et al., 2010	African American and whites in Baltimore 3720 participants	Started in 2009 Status: Ongoing (Wave 5 of follow-up) https://clinicaltrials.gov/ct2/show/NCT01323322
The NIA’s Baltimore Longitudinal Study of Aging	Comprehensive and longest running longitudinal examination of human aging in the world. The aim was the understanding about normal versus pathological aging as well as age-related diseases and conditions.	Schrack et al., 2014	Different ethnicities** 3200 participants (1300 actively followed)	Start date: 1958 Status: Ongoing https://www.nia.nih.gov/research/labs/blsa
Framingham Heart Study	Long-term cardiovascular cohort study of residents of the city of Framingham, Massachusetts, aimed at understanding epidemiology of coronary heart disease (CHD)	Mahmood et al., 2014	Different ethnicities** Six groups of individuals: Original Cohort (5209), Offspring Cohort (5124), Third Generation Cohort (4095), New Offspring Spouse Cohort (103), Omni Generation 1 Cohort (506), and Omni Generation 2 Cohort (410).	Started in 1948 Status: Ongoing https://framinghamheartstudy.org/
Lifestyle Interventions and Independence for Elders (LIFE) study	Study of the effect of physical activity in reducing the risk of major mobility disability.	Pahor et al., 2014	Different ethnicities** 1635 participants	Started in 2010 Status: Completed https://clinicaltrials.gov/ct2/show/NCT01072500
Genetics of Healthy Aging (GEHA)	EU-funded program on the identification of genes involved in healthy aging and longevity.	Skytthe et al., 2011	Individuals of European ancestry from 10 recruitment areas all over the Europe 2535 90+ sibpairs (5319 non-agenarians) 2548 controls (50–75 years)	Started in 2004 Status: Completed (2007) https://cordis.europa.eu/project/id/503270/it
IDEAL (Integrated research on Developmental determinants of Aging and Longevity)	EU-funded project on development, epigenetics and longevity. The project was focused on gathering insights into the role of early life conditions affecting late-life health, disease and aging.	Deelen et al., 2013	Individuals of European ancestry from all over the Europe 8,000 long-lived individuals (≥85 years of age)	Started in 2011 Status: Completed (2016) http://www.idealageing.eu/
Canadian Longitudinal Study on Aging (CLSA)	Large, national, long-term study aimed at understanding the impact of biological, medical, psycho-social, lifestyle and economic factors on the development of disease and disability as people age.	Raina et al., 2009	Canadians 50,000 individuals aged between 45 and 85 years followed up for 20 year at least	Started in 2003 Status: Ongoing https://www.clsa-elcv.ca/
Nagahama Prospective Cohort for Comprehensive Human Bioscience (the Nagahama Study)	Longitudinal cohort study of the residents in Nagahama City (south-central Japan)	Matsumoto et al., 2017	Japanese 9,764 participants at baseline aged between 34 and 80 years	Started in 2013 Status: concluded (2016) no website

N.B: *Number of Samples: number in parenthesis indicates the number of samples collected in each cohort. **Different ethnicities in United States studies generally include: Hispanic, White, African American, Asian, Others.

Together with the collection of such large cohorts, including big data of phenotypes related to aging, technical advancement and the lowering of costs of genome-wide genotyping and next-generation sequencing technologies prompted the discovery of new genetic variants associated with aging. This has increased the number of databases devoted to host big-data generated by the large number of genome-wide association studies (GWAS) launched on human age-related diseases (International Mhc and Autoimmunity Genetics Network, Rioux et al., 2009; Márquez et al., 2018; Jansen et al., 2019).

Meta-analyses of GWAS, carried out by combining the results of independent studies (called cross-disease meta-analysis), trying to identify loci with both same-direction and opposing-direction allelic effects, revealed significant heterogeneity of disease association within the genome, although some regions showed association with more than one diseases (*P* < 0.0001) (Jeck et al., 2012). Loci with pleiotropic effects on age-related disorders tend to be enriched in genes involved in underlying mechanisms related to nervous, cardiovascular and immune system functions, stress resistance, inflammation, ion channels and hematopoiesis, supporting the hypothesis of shared pathological role of infection, and inflammation in chronic age-related diseases (He et al., 2016).

Notwithstanding, there has been a lack of replication when comparing these studies, due to differences in sample size, study-specific age cut-offs to define the affectation status, sex-specificity, and population specificity, i.e., genetic and/or lifestyle heterogeneity among cohorts.

Nowadays, successful insights in the complex field of studying the human aging can be generated only by large multidisciplinary groups, because of the gerontological research need to bring together a large number of sources of information: these are essential to better understand how genetic and environmental components interact, and result in different health outcomes in older adults.

## Data Integration in Aging Studies: Needs and Challenges (of Omics)

The advancement of many technologies has made omics sciences (genomics, transcriptomics, proteomics, and metabolomics) increasingly affordable. The use of next-generation sequencing, and the versatility of this technology, has paved the way for data integration: through the same technology, it is today possible to investigate genetics (targeted, exome and whole-genome sequencing), different aspects of genomics like conformational capture (Hi-C/3C-Seq) or protein binding (ChipSeq), and interrogate the transcriptome (RNAseq) (Williams et al., 2015). The consequence of this technological convergence is that different information can be represented by similar data formats and data sources: this offered unprecedented opportunities for further development of the -omics and a boost to developing new integration methods and approaches. Aging research is perhaps one of the subjects where data integration is becoming essential to further the understanding of this trait: as mentioned above, long-living individuals have escaped the major causes of death, and therefore their phenotype could be considered as complementary to a large number of complex pathological phenotypes. The most complex, among complex traits, we could say. Additionally, if we look at the genomics determinants of human aging, long living individuals are likely to be phenocopies, i.e., where different polygenic combinations result in the very same phenotype. Such a phenomenon severely impacts on our capacity to unmask not just the underlying molecular mechanisms, but the necessary interplay between all genes involved and their genetic variation in order to produce the trait we observe phenotypically (Lescai and Franceschi, 2010).

For this reason, aging is considered a multi-factorial trait, highly heterogenous from a genomics point of view, characterized by different levels of complexity ranging from molecular to cellular, organ and organism (Cevenini et al., 2010): in order to be investigated properly, this complexity requires a systems-biology and -omics approach where the integration of multiple data becomes essential.

Describing a biological phenomenon by investigating multiple aspects of the biology or pathophysiology at the same time has become not only easier, but also more accessible. Big data is breaking down traditional boundaries between fields: collecting such larger datasets also means to integrate data generated through the use of different approaches (for instance, both genome wide and family study design). This also implies the involvement of hundreds of thousands of individuals over many decades, to study the effects of earlier life conditions on later-life health, including genetics, behavior and contextual factors such as socioeconomic status.

Single-cell analyses have provided an additional dimension to investigate the complexity of the organisms: technology allows now to overcome the “average” picture we get from whole tissues, and investigate the genomics, transcriptomics, proteomics and metabolomics of each single cell analyzed. There is a growing interest in the application of this technology in aging studies as well, although most data available in Humans are limited to transcriptomics (Uyar et al., 2020). Other single cell -omics data emerging in model organisms show the potential of this application and the importance in this field for the generation of new in-depth data on aging biology (He et al., 2020).

Generating more data, however, means that the additional information has to be integrated, in order to offer a rational insight into the biology, and an answer to the experimental question: one suddenly has to deal with several layers of complexity. Next generation sequencing is indeed a very powerful tool to address also genetic heterogeneity in traits, and therefore does help investigating complex phenotypes. Data integration remains, however, a very challenging task, because of its mathematical and statistical nature, but also due to costs and experimental difficulties: it is often quite difficult to use the same set of samples in order to collect all different types of data, and therefore integration methods need to be able to handle the resulting gaps in information.

Multi-layered networks for example have been proposed as a powerful tool used to establish the necessary connection between different types of information: it does provide a natural way to represent the structure of a biological system, and the relationships between different layers in the network may represent effects which cannot be described just by statistical correlations (as it happens in genome-wide association studies, GWAS) (Lee et al., 2019). Network-based methods appear also a very appropriate direction to combine data integration tools with a holistic interpretation of phenotypes and their determinants. It is for example through network analysis, that Garcia Alonso et al. (2014) have proposed a mechanism for the maintenance of deleterious variants in the genome of Human populations: by looking at the whole interactome, we are able to better understand how deleterious mutational load can be suppressed in the resulting phenotype (Garcia Alonso et al., 2014). Similarly, Khurana et al. (2013) have used a network approach to aid the interpretation of genomics variants. Multi-layered networks seem to offer also a promising solution to some data integration challenges of single-cell omics analyses (He et al., 2020).

Tensor decomposition has also been proposed as a quite powerful method to infer relationships between different biological descriptors. A tensor is a multi-dimensional array: the decomposition of these higher order arrays had numerous applications in a wide ranges of scenarios, but only recently found interesting applications in biology, thanks to the increase in data dimensionality. There are a few methods to decompose higher-order tensors (PARAFAC, Tucker among others) (Kolda and Bader, 2009), and they can be considered a generalization of more widely known methods used in biology like singular value decomposition (SVD) or principal component analysis (PCA). They can be very powerful in discovering patterns in the data, and uncovering hidden relationships, as well as in providing a joint factorization of multiple data sets, which is a key issue in data integration (Khan et al., 2016). This kind of methods has been applied as a way to investigate expression in multiple tissues, and in linking transcriptomics patterns to genetic variation (Hore et al., 2016), or to integrate genomic and epigenomic data (Fang, 2019). Another area where tensor decomposition has been successfully applied is data visualization: it is becoming increasingly important, to provide a much better way to explore, and consequently understand, high-dimensional datasets and multi-omics data. Projections of the lower-dimensional decompositions allow unmasking hidden patterns, finding new relationships and applying clustering methods otherwise inaccessible to higher-dimensional data (Fanaee-T and Thoresen, 2019).

Machine learning (ML) approaches have also proven to be extremely powerful in the re-analysis of large datasets collected in the past, allowing an unprecedented capacity for data integration, and providing new insights. It is the case, for example, the use of feature selection and a combination of support vector machines (SVM) and random forest (RF) allowed to mine the combined datasets of different aging population studies (3C, 3-City; AMI, aging multidisciplinary investigation; TSHA, Toledo Study for Healthy Ageing; InCHIANTI, Invecchiare in Chianti), and enabled the integration of lifestyle, laboratory and clinical data. This approach allowed the processing of more than 30 thousand omics markers, confirming and expanding the understanding of mechanisms involved in frailty (Gomez-Cabrero et al., 2021). The study also provided an important starting point for future studies in the field.

## Omics Bioinformatics Useful for Aging Research

Bioinformatics has evolved dramatically in the past 10 years. This has definitely affected aging research as well. Nowadays, there is a strong drive for bioinformatics solutions to adopt at least three key principles: reproducibility, portability, and community standardization.

Reproducible research has been a goal for many years (Gentleman et al., 2004; Gentleman, 2005) and it has been facilitated by a number of solutions in data science, thanks particularly to the possibility of mixing comments, text, and blocks of code together. Reproducibility however is not achieved just by sharing the code used for the analysis, or by explaining in a transparent way how it has been written. Furthermore, it is achieved by documenting the workflow of activities in their specific sequence of tools used for the analysis, keeping track of the software versions, of the provenance of files and enabling any other user to access and run the very same sequence of data analysis tasks. The latest developments in domain specific languages (DSL) dedicated to running analysis workflows in the life sciences have certainly changed the way biological scientists approach bioinformatics: it has become easier to build and run and share reproducible workflows, but they have also become more accessible to people who are not necessarily experts in bioinformatics. In our work we have adopted one of such DSL, namely Nextflow (Di Tommaso et al., 2017), which is certainly having a major impact in data analysis for life sciences, and aging research as well.

It has certainly been more challenging in areas which involve a large amount of experimental work, where reproducibility also implies transparency and accessibility to reagents, source of materials and methods for their collection. Biological research has more recently seen a major effort to overcome these challenges (Lithgow et al., 2017), also in gerontological sciences (Estabrook, 2020).

Reproducibility is certainly connected to the concept of portability, which addresses another major challenge of bioinformatics: the possibility of running the same workflow, independently of the computing environment, infrastructure, or location of the computing resources (for example, on-premise or on-cloud). The integration of workflow managers with the increasingly adopted container technology (Docker, Singularity), or recipe-based packages (Anaconda) has provided a solution to both reproducibility of software as well as to the portability challenge (Di Tommaso et al., 2015).

Reproducibility and portability represent fundamental characteristics of a bioinformatics pipeline, but standardization is also an essential goal. Standards can be either regulated, as it happens in some areas, or they become really valuable when they are developed and adopted by a community of practice. This is most likely the case in bioinformatics and data science. A very original effort has been made around the use of workflow managers, and a community in particular, built around the use of Nextflow, is worth of notice: the nf-core community (Ewels et al., 2020). Those defined as “community curated pipelines” have become *de facto* community standards for bioinformatics, and address key applications ranging from RNAseq to WGS, to metagenomics. This initiative is having an influential impact for two reasons: first, the pipelines are formulated through a collaborative effort in a lively community, resulting in the adoption of solutions which respond to the latest published best practices in each field; second, this community is also providing a set of templates, and co-developed code guidelines, which increase accessibility to these tools, and provide a great environment for people to start from, thus mitigating the learning curve in the adoption of workflow managers.

The bioinformatics challenges described above remain valid for many areas of science, including aging research. The understanding of this phenotype, however, involves particular challenges in the area of computational modeling: it is through modeling that a connection can be found, among the many elements underlying the biology of aging. One could therefore use workflow languages like Nextflow, in order to process raw data, perform initial data integration and network-based analysis, add classical pathway-based analyses (Zhao et al., 2018), and then follow-up with appropriate tools designed for modeling biological systems.

These include biochemical modeling tools like CellDesigner^1^ (Funahashi et al., 2003) for gene-regulatory and biochemical networks, COPASI^2^ also meant for the simulation of biochemical networks and their dynamics, but also more generally valid tools like the systems biology mark-up language (SMBL) (Hucka et al., 2018), which has been successfully used to model higher-order brain dysfunctions (McAuley et al., 2009). It is also by computational modeling that it was proposed how the decline we usually observe in some physiological processes during aging, might act like a “programmed deterioration” in order to increase the efficiency of other functions (Markov et al., 2018). Unfortunately, as intriguing as the hypothesis is, the same model fell short of identifying appropriate mechanisms and aging genes as observed in populations. Similar approaches, however, applied on the integration of omics data in model organisms like *Caenorhabditis elegans*, succeeded in improving the understanding of the contribution of different -omics data to the overall characterization of an organism (i.e., how they play a different role in sample variability), and also in proposing a unifying hypothesis to connect the metabolic switches observed during aging (food intake, among others) and the drop in mitochondrial function (Hastings et al., 2019).

## Large Data Collections for Aging: A Survey of Available Databases and Datasets

**FIGURE 1:**
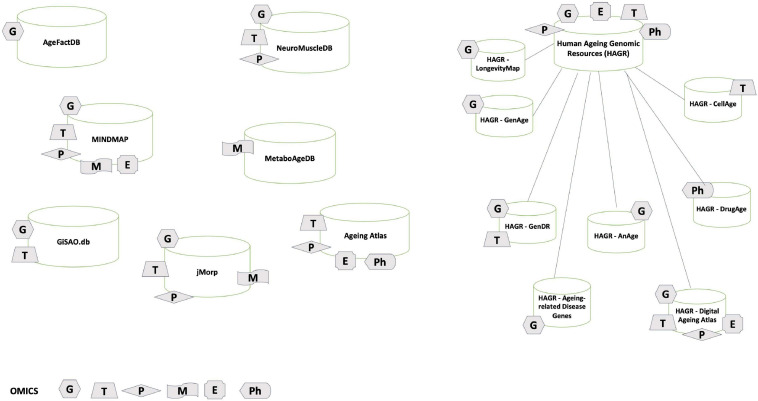
Omics aging databases. General overview of the main aging research databases described in the review: we have annotated each database with the omics data type it provides. G stands for genomics; T for transcriptomics; P for proteomics; M for metabolomics; E for epigenomics; Ph for pharmacogenomics.

Nowadays, efforts pruned to collect data on aging phenotype provide us several databases useful to integrate data and analyze the biological pathways implicated in the aging process (Figure 1).

In Table 2 we have compiled a list of useful databases in aging research, which we hope the reader will find a useful resource to access results and metadata. Common characteristic to all the databases is the integration of different data sources, with common identifiers linking to NCBI and establishing the connection with scientific literature, and sometime meta-analysis of studies in the field. In most cases, the interfaces are user-friendly and they allow data download in different formats.

**TABLE 2:** Aging research databases.

Database	Brief description	Data type and size	Omics data	Project status	References	Links
AgeFactDB	Database for the collection and integration of age-related data	16,599 aging factors (16,450 genes, 91 compounds, 58 others) and 9,611 observations (8,159 aging phenotypes, 1,452 homology analyses)	Genomics	Stopped	Hühne et al., 2014	http://agefactdb.jenage.de/
MINDMAP	Integrated database for research in aging, mental wellness, and urban environment. It integrates 10 longitudinal cohort studies across cities in Europe, the US, and Canada to investigate mental wellbeing in older age as well as age-related factors and phenotypes	Aging factors, aging-related phenotypes, and aging-related risk factors in 2,664,115 participants	Genomics, Epigenomics, Transcriptomics, Proteomics, Metabolomics	Ongoing	Beenackers et al., 2018	http://www.mindmap-cities.eu/
GiSAO.db	Database of genes involved in age-related biological processes. It also contains orthologs between *Homo sapiens*, *Mus musculus*, *Saccharomyces cerevisiae*, *Caenorhabditis elegans*, and *Drosophila melanogaster*	Data of genes involved in senescence, apoptosis, and oxidative stress (gene expression data, annotation data, experimental data, ortholog data) for a total of 338 between experiments and arrays performed on all species involved	Genomics, Transcriptomics	Stopped	Hofer et al., 2011	https://gisao.genome.tugraz.at/
NeuroMuscleDB	Database of muscle-related genes at different stages of development and aging	Information of about 1,102 genes, 6,030 mRNAs, and 5,687 proteins that participate in muscle development in *Homo sapiens*, *Mus musculus*, and *Bos taurus*	Genomics, Transcriptomics, Proteomics	Ongoing	Baig et al., 2019	http://yu-mbl-muscledb.com/NeuroMuscleDB/
MetaboAgeDB	Database of human aging-related metabolites	408 annotated aging-related metabolites and more than 1,515 aging-related variations occurring in healthy individuals	Metabolomics	Ongoing	Bucaciuc Mracica et al., 2020	http://www.metaboage.info/
Human Aging Genomic Resources (HAGR)	Collection of databases and tools for studying the genetics of aging	It integrates 10 between databases and tools related to genomics, 1 related to drugs, 1 to animal longevity, and 1 to aging changes	Genomics, Epigenomics, Transcriptomics, Proteomics, Pharmacogenomics	Ongoing	Tacutu et al., 2018	https://genomics.senescence.info/index.php
HAGR - GenAge	Database of genes related to aging in model organisms and in humans	2,202 genes related to longevity and/or aging in model organisms, and 307 aging-related genes in humans (both directly related to aging in humans and the best candidates from model organisms)	Genomics	Ongoing	{Tacutu:2018fua}	https://genomics.senescence.info/genes/
HAGR – GenDR	Database of genes associated with dietary restriction (DR) in model organisms and in mammals	214 DR-associated genes in model organisms and 173 differentially expressed genes due to DR in mammals	Genomics, Transcriptomics	Ongoing	Wuttke et al., 2012	https://genomics.senescence.info/diet/
HAGR – LongevityMap	Database of genes, genetic variants, and loci associated with longevity in humans	550 entries (275 reported as significant findings), 884 genes, and 3,144 variants from a total of 270 large and small-scale association studies on longevity in humans	Genomics	Ongoing	Budovsky et al., 2013	https://genomics.senescence.info/longevity/
HAGR – CellAge	Database of genes associated with cell senescence	1,259 cell senescence-associated gene expression changes from 279 gene manipulation experiments for 164 distinct cell lines and 3 distinct senescence types	Transcriptomics	Ongoing	Avelar et al., 2020	https://genomics.senescence.info/cells/
HAGR – Aging-related Disease Genes	Dataset of genes involved in age-related diseases	769 aging-related disease genes	Genomics	Stopped	Fernandes et al., 2016	https://genomics.senescence.info/diseases/
HAGR – DrugAge	Database of drugs and compounds associated to extended longevity in model organisms	567 distinct compounds across 1,823 lifespan (increasing or decreasing) assays on 30 unique species	Pharmacogenomics	Ongoing	Barardo et al., 2017	https://genomics.senescence.info/drugs/
HAGR – AnAge	Database of longevity records in animals	4,244 entries (4,219 species and 25 taxa) with 3,275 longevity records, and 1,981 aging process observations. Life history traits for 3,275 species and metabolism data for 707 species	Genomics	Ongoing	(Tacutu et al., 2018)	https://genomics.senescence.info/species/
HAGR - Digital Ageing Atlas	Database consisting in a collection of human age-related data covering different biological levels. It also contains data on *Mus musculus*	3,784 molecular changes (3,071 in humans, 713 in mice), 343 physiological changes, 17 psychological changes, and 95 pathological changes in humans. A total of 2,599 genes involved for humans and 675 for mice	Genomics, Trancriptomics, Proteomics, Metabolomics	Ongoing	Craig et al., 2015	http://ageing-map.org/
Aging Atlas	Database of age-related changes and pathologies in humans and model organisms	3,274 aging-related human and mouse genes. RNA-seq data of genome-wide transcriptomic changes related to aging (more than 18,000 differentially expressed genes potentially related to aging). Single-cell RNA-seq data from 14 types of aged tissues from rats, monkeys, and humans. ChiP-seq data of specific aging-related loci regulated by histone modifications and transcription factors. Protein–protein interaction data related to aging. Compounds related to aging	Genomics, Epigenomics, Transcriptomics, Proteomics, Pharmacogenomics	Ongoing	Aging Atlas Consortium, 2021	https://bigd.big.ac.cn/aging/index
Japanese Multi Omics Reference Panel (jMorp)	Database of metabolome and proteome data in plasma obtained from volunteers in Tohoku Medical Megabank Organization. It also integrates other multi-omics data collected from volunteers mainly from Japan	A Japanese reference genome and genomic data from 8,380 Japanese individuals. Cell-type specific transcriptomes based on 100 Japanese individuals. Peptides of 256 abundant proteins in 501 volunteers. 45 metabolites detected in 25,783 individuals	Genomics, Transcriptomics, Proteomics, Metabolomics	Ongoing	Tadaka et al., 2021	https://jmorp.megabank.tohoku.ac.jp/202102/

The “**AgeFactDB,**” the JenAge Aging Factor Database^3^ is a repository aimed at the collection and integration of aging phenotype data including lifespan information (Hühne et al., 2014). Goal of the database is focusing on ‘Aging Factors.’ AgeFactDB incorporates information on genes, chemical compounds, environmental or lifestyle factors such as diet, whose action can affect lifespan and/or another aging phenotype. In order to accept an aging factor in the database, a comparison of two different experimental setups (e.g., experiments with and without a chemical compound, a variation of concentrations, dietary restriction or overfeeding vs. a normal diet, etc.) is required. When a factor is defined, each information linked to the effects of that aging factor is called ‘Observation’ and represents aging-related evidences. The AgeFactDB provides a unique ID to identify aging factors and observations (“AF_nnnnnn” and “OB_nnnnnn,” respectively).

A huge issue in data integration is usually represented by the *different data structures* of the originating data sources: they have to be integrated with a large manual curation effort. AgeFactDB attempts at solving this challenge, by providing aging phenotype information in two formats: Type 1, which includes observations un-separated within a single description, Type 2 containing lifespan data in separate fields (e.g., lifespan effect, lifespan change, and lifespan value). Another major issue for all databases is the *data validation*. Comparing a list of observations sorted by the lifespan change given in %, to the qualitative classification (increased, decreased, and no statistically significant effect) AgeFactDB is able to identify a number of inconsistencies. AgeFactDB can be accessed either by browsing through predefined lists or by searching, using as queries synonyms, PubMed IDs and Medical Subject Headings, choosing among more source databases or with specific type(s) of aging-relevant evidence or search by using AgeFactDB ID. More details are available in Hühne et al. (2014).

The **MINDMAP**^4^ is an integrated database infrastructure for the promotion of research in aging and the management of mental well-being and cognitive function of older individuals (Beenackers et al., 2018). Mental disorders in old age are related to impairments in the ability to function socially, decreased quality of life, and increased risk of health problems and comorbidities, thus they are considered a key priority for public health policy and prevention (Whiteford et al., 2013). The aim of the database is to integrate urban environmental characteristics linking together longitudinal studies from 11 countries covering over 35 cities. Integration of these data is useful to evaluate the interaction between environment and individual determinants of cognitive aging. The strength of the MINDMAP is the capability to combine data from multiple cities and from different sources (physical, social and socioeconomic environmental characteristics, policy indicators), and therefore the opportunity to increase sample sizes and statistical power, essential to identify high-risk population subgroups and to study relatively rare health conditions. Like for other databases, the *harmonization of data derived by different studies* remains a key challenge: to harmonize all MINDMAP cohort studies, each research team works on a specific domain of information (e.g., socioeconomic variables, multi-morbidities, health behaviors variables, etc.). The database has restrictive data sharing rules, so that a central server running RStudio allows authenticated investigators to securely access firewall-protected data on primary and secondary data servers. More details are available in Beenackers et al. (2018).

**NeuroMuscleDB^5^** is a database of genes associated with muscle development, neuromuscular diseases, aging, and neurodegeneration (Baig et al., 2019).

The aim of the database is to help in developing strategies to contrast muscle loss in elderly, i.e., one of the major contributors of neuromuscular diseases and neurodegeneration which affects mortality in old age (Listrat et al., 2016). A goal of this resource is to help in translating the findings of different studies into clinical interventions. Thus, NeuroMuscleDB integrates results coming from muscle-associated genes directly or indirectly involved in aging and age-associated neurodegenerative diseases. The database can be manually or systematically updated, by incorporating new data and resources. A strength of this database is that analytical tools, containing PCR primer design and sequence analysis, were also implemented to support the laboratory analyses of candidate genes and sequences. More details are available in Baig et al. (2019).

Molecular studies on metabolic variations during aging can henceforward guide lifestyle changes and/or medical interventions directed to improve healthspan and lifespan (Lorusso et al., 2018). Although the research of aging is a rapidly emerging field, none of the available aging-related databases is specialized in aging metabolomics. **MetaboAgeDB^6^** is a source of known age-related metabolic changes from studies of disease-free human cohorts (Bucaciuc Mracica et al., 2020). Aging-sensitive metabolites, extracted from well-known databases, are annotated with their chemical information, variations between age groups, linked to the metabolic pathways in which they are involved, including their effect on ageing and the gender(s) in which this effect can be specifically seen. This is obtained thanks to a quick link to individual pages including an ‘Age-variations’ panel, in which gender-specific and method-specific metabolite variations are visually represented, grouped by the type of age-related variation. For each metabolite, a summary table with an overview of the information on the units of measurement, the method by which the metabolite is detected, the age range and sex of experimental group as well as information about specific pathways that the metabolites are involved in are available. In addition, MetaboAge entry provides users to use external links, through an easy and user-friendly web-interface. More details are available in Bucaciuc Mracica et al. (2020).

The Human Ageing Genomic Resources (HAGR)^7^ is a collection of databases and tools designed to help researchers interested in the genetics of human aging, integrating results from different approaches such as functional genomics, network analyses, systems biology and evolutionary analyses. The project is supported and maintained by the Integrative Genomics of Ageing Group at the University of Liverpool in the United Kingdom. Such big data repository is divided in sections, which will be shortly indicated below.

A major resource in HAGR is **GenAge^8^**, the database of genes related to longevity. Its main characteristic is the division in two sections, i.e., the section on human aging-related genes includes the few genes directly related to aging in humans plus the best candidate genes obtained from model organisms (yeast, worms, flies, mice, etc.), clustered according to functional groups (Tacutu et al., 2018). At the time of the last update, February 2020, the database included 307 human genes, belonging to 15,054 Gene Ontology categories. The developer of GenAge claim to be the first to construct and analyze a protein network of human aging as well as develop a system-level interpretation of aging.

Complementary to GenAge is **LongevityMap**, a database of human genetic variants associated with longevity^9^, a repository of genetic association studies of longevity which includes both positive and negative association results, to provide visitors with as much information as possible regarding each gene and variant previously studied in context of longevity. Searching the LongevityMap can be done by chromosome, by gene or genetic variant (e.g., refSNP number), entering gene’s name or use the gene’s HGNC symbol, or through a topic, like an age-related disease, in **LibAge^10^**. LongevityMap provides the link to **AnAge^11^,** the Database of Animal Aging and Longevity, a repository developed for comparative biology studies, to provide researchers with quantitative data for applying the comparative method to studies of life history and lifespan. The database, featuring over 4,000 species, contains life history records of organisms, accessible to the AnAge’s browser and divided in three branches (kingdom of animals, plants and fungi). The most important trait in AnAge is maximum longevity (also called maximum lifespan) because it is the most widely used parameter for comparing rate of aging between species. Factors which can bias longevity records, such as population size and whether animals are kept in captivity or not, are also considered. Each entry has a qualifier of the confidence placed in the longevity data. This qualifier is based on the reliability of the original reference from which maximum longevity was obtained, sample size, whether a given species has been studied and reproduces in captivity, and whether there are any conflicting reports. Confidence in the longevity data is hence classified as: ‘low’ (only used for species without an established maximum longevity in AnAge), ‘questionable,’ ‘acceptable,’ and ‘high.’ The database can be interrogated or it is possible to download a zipped tab-delimited dataset of the latest stable build, containing only the raw data, not observations.

**GenDR^12^**, **DrugAge^13^,** and **CellAge^14^** are other resources accessible from HAGR. GenDR is a database of genes associated with dietary restriction. Like CellAge, the database of human senescence-associated genes, classify genes on the base of genetic manipulation experiments and gene expression profiling. Dietary restriction (DR), limiting nutrient intake from diet without causing malnutrition, is the most reproducible way to extend lifespan in multiple organisms and postpone age-related degeneration. GenDR includes two datasets: (1) genes inferred from experiments in model organisms in which genetic manipulations cancel out or disrupt the life-extending effects of DR; (2) genes robustly altered due to DR, derived from a meta-analysis of microarray DR studies in mammals, including also an analysis of the gene network. Understanding the genetic basis of DR is of great importance not only to the biology of aging but also to understand how diet can influence aging, longevity, health and age-related diseases. In particular, pharmaceutical interventions targeting DR-associated genes are an emerging area with huge potential. In this frame, DrugAge provides data on over 500 drugs, compounds and supplements (including natural products and nutraceuticals) with anti-aging properties that extend longevity in model organisms. CellAge also annotates 279 human genes driving cellular senescence, and allows to specifically browse genes associated with cellular senescence, simply by querying if a gene of interest is associated with cell senescence in animal models, and to search for molecular signature, i.e., genes that are either over-expressed or under-expressed during replicative senescence of human cells. The base of the work contained in this database is that genes involved in cellular senescence tend to be overexpressed with age in human tissues and are significantly overrepresented in anti-longevity and tumor-suppressor genes, while genes inhibiting cellular senescence overlap with pro-longevity and oncogenes. Furthermore, cellular senescence genes are strongly conserved in mammals but not in invertebrates.

By integrating the above mentioned and other datasets, Avelar et al. (2020) recently developed a multidimensional analysis of cellular senescence. By studying protein–protein interaction and co-expression networks, the researchers found an enrichment for cell cycle and immunological processes among the senescent regulators; by siRNA silencing, they prompted 13 genes (C9orf40, CDC25A, CDCA4, CKAP2, GTF3C4, HAUS4, IMMT, MCM7, MTHFD2, MYBL2, NEK2, NIPA2, and TCEB3) able to decrease cell number, activate p16/p21 pathway, and undergo morphological changes resembling cellular senescence.

Finally, HAGR links also to **The Digital Aging Atlas^15^** a centralized collection of aging changes and pathologies. Maintained by the Aging Atlas Consortium (Aging Atlas Consortium, 2021), the database integrates molecular, physiological, psychological and pathological age-related data, including anatomical models. Although primarily focused on human aging, the db also includes supplementary mouse data, in particular gene expression data, to enhance and expand the information on human aging. The genetic information maintained in the DB is also quite relevant, with 2,599 Human genes and 675 Mouse genes, linked to age-related diseases or traits.

A further resource available for researchers in the field is offered by Aging Analytics^16^, a very large repository of information on longevity and aging, maintained by a non-commercial and no-profit Deep Knowledge Group. The aim of this repository is to offer progress updates on these topics, ranging from publications, to newly identified biomarkers, to research groups working in this area. The website also lists biotech companies available for consultancy in aging research.

Besides the information organized in existing databases, we aimed at providing readers with a carefully curated reference to other available data sources. In Supplementary Table 1, we report a selection of datasets from online repositories, resulting from age-related studies producing different omics data. We have searched and examined one by one the results and extracted those we believe most relevant in this context.

The first repository we scanned for relevant data has been the database of Genotypes and Phenotypes (dbGaP^17^). DbGaP includes data from sequencing studies and large-scale genomic studies, as well as genotype, phenotype, exposure, expression array, epigenomic, and pedigree data from GWAS. The access to data hosted on dbGaP is achieved by signing in to the authorized-access portal and submitting an application for specific datasets. Requests must be reviewed and then approved by a specific data access committee (DAC). We performed a search in the dbGaP database on aging-related genetic studies. The aim was to find and select the main datasets that one could access/request to perform omics studies. We included in Supplementary Table 1 the large scale GWAS studies and omics studies related to aging. We found and selected 26 dbGaP studies. The majority (17) applied genomics approaches, either involving whole-genome or targeted sequencing (7), in few instances in parallel with whole-exome sequences, or whole-genome genotyping (10). Two studies combined single nucleotide polymorphism (SNP) arrays with exome sequencing. Eight were exclusively based on genome-wide genotyping, employing different arrays with different numbers of SNPs. However, care must be taken in the interpretation of these data due to ascertainment bias and to the fact that rare alleles can be under-represented in these arrays. Indeed, most SNPs used by commercial arrays were ascertained in European populations (Albrechtsen et al., 2010; Lachance and Tishkoff, 2013). As a result, disease risks can be mis-inferred and not yield accurate estimations depending on populations, highlighting the need of taking into account ancestry of study participants. Moreover, cost-effective alternatives to genotyping arrays, such as low-coverage sequencing (≥4X), have been shown to capture variants at all frequencies more precisely and to identify novel variation in underrepresented populations, as Africans (Martin et al., 2021). The rest of the studies we have included (9) are based either on a single or on more omics technologies. In particular, they were based on transcriptomics (RNA sequencing) and/or epigenomics (5hmC capture sequencing, DNA methylation, ATAC sequencing, ChiP sequencing). Few of them combined more omics approaches: those datasets would allow for a step forward toward multi-omics data integration, because they are among the few based on the same initial sample sets. Neither proteomics nor metabolomics data were found in this search.

In Supplementary Table 1, we also report the results of the same search on the European Genome-Phenome Archive (EGA^18^). The EGA is an online repository for the storage and sharing of genetic and phenotypic data from biomedical studies. Studies present on EGA consist of one or more datasets, each one under the supervision of a DAC. To access data on EGA, an application must be sent for each dataset of interest to the respective DAC, which will review and approve the request.

We searched the EGA repository for aging-related genetic data that can be used to perform multi-omics studies and data integration. We found many studies whose pages and datasets from EGA referred to dbGaP pages, and whose data were deposited on dbGaP. Therefore, the relevant studies were either already included in our selection from dbGaP or were added to it. For proper EGA projects and datasets, we selected 11 studies. Genomics (whole-genome and whole-exome sequencing), epigenomics (DNA methylation, MeDIP sequencing, and ATAC sequencing), and transcriptomics (RNA sequencing) were almost equally represented (four, four, and three studies, respectively). Only one project combined two omics technologies, whereas we did not find any multi-omics study. As in dbGaP, neither proteomics nor metabolomics data were present.

For those who wish to perform a broader search on omics datasets, we would recommend OmicsDI^19^ (Perez-Riverol et al., 2017, 2019). This web-based tool connects a very large number of resources, listing omics studies and omics datasets. The results of a search for the terms “ageing” or “aging” can be overwhelming, and often after careful inspection no actual datasets can be found, either with public or gated access. Nevertheless, it is an interesting tool to keep in mind, with the caveat that some time has to be spent in reviewing its results.

## Post Genomics: Perspectives in Bioinformatics

Advances both in high-performance computing as well as in ML methods and in particular deep learning approaches, have scaled up the opportunities to integrate different data types. Deep learning methods have the advantage of building hidden layers, which learn features capable to best predict given outcomes: this allows the identification of novel patterns in very complex datasets, and provide a very powerful tool for biology applications, able to extract predictive parameters even from very complex datasets. With this in mind, these approaches could provide more holistic and system-base views of a biological system, and therefore offer a deeper understanding of biological mechanisms driving any phenotype (Hudson, 2021).

An interesting example is provided by the use of deep neural networks (DNN) on a large set of biomarkers available through common blood testing: Putin et al. (2016) used an ensemble of DNNs and trained them on biochemical parameters from 62,419 individuals, achieving and interesting performance in the prediction (*R*^2^ = 0.8). More interestingly, this approach allowed feature extraction, and identify albumin, glucose, alkaline phosphatase, urea, and erythrocytes as most promising markers for predicting human chronological age (Putin et al., 2016). This exercise showed the data mining potential of these new methods, even in well-investigated areas like biomarkers.

**FIGURE 2:**
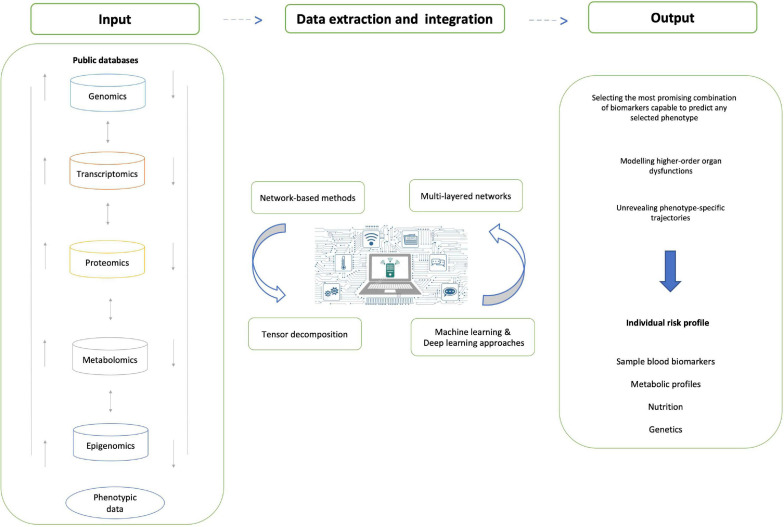
Data integration in aging research. A schematic representation of the process of data integration from public databases and other sources in aging and age-related diseases. The main data sources are represented in the “input” panel, and we represent the key methods described in the manuscript under “data extraction and integration.” We have represented the expected answers in the output, in terms of risk profiles and predictive tools for population stratification and prevention.

Feature selection is in fact a critical and actionable area when considering potential application of -omics sciences: while large-scale omics data are essential to provide insights into the aging phenotype, a selected number of actionable elements has to be identified in order to enable strategies for intervention. Galkin et al. (2020) also used deep learning (DL) to predict chronological age, but trained their algorithms on the taxonomic profiling of Human gut microbiomes: a strategical choice, considering the growing importance attributed to the microbiota, and in turn on nutrition for wellbeing and health. Leclercq et al. (2019) while following a similar approach, chose to develop a software, called BioDiscML, which makes use of different ML algorithms to select the most promising combination of biomarkers capable to predict any selected phenotype. They showed how this software can be applied in a variety of real-world datasets, including stem cells, nervous systems tumors and prostate cancer: a key aspect of this work has been the attempt to break the non-expert’s barrier often represented by the use of ML and DL algorithms. The trend shows that, while bioinformatics continues to advance the field, and more powerful methods are proposed and tested, existing methods become more and more accessible, and therefore have progressively more impact on daily choices both in research as well as in intervention strategies (Figure 2).

We have compiled in Table 3 a useful list of tutorials, which readers can use as a starting point to approach the key tools and methods we discussed in this review.

**TABLE 3:** Suggested tutorials about bioinformatics methods and tools for omics analyses.

Topic	Description	Links
Tucker Decomposition	This is an interesting blog curated by a company named “Integrated Knowledge Solutions.” We found this 3-parts tutorial well explained and accessible, and therefore a good starting point for those wishing to approach tensors and Tucker decomposition using R	Part 1: https://bit.ly/2PEb5KC Part 2: https://bit.ly/3dfNrMK Part 3: https://bit.ly/39pxjXR
Tucker Decomposition	R-bloggers is a famous blog for those who use R, where people contribute with their expertise and release tutorials on different topics. Here, Alexej Gossmann nicely explains the basic concepts of tensors, and how to perform Tucker decomposition	Understanding tensors: https://bit.ly/3czBK4t Tucker decomposition: https://bit.ly/3rAWVHo
Support Vector Machines	scikit-learn is a reknown python framework to carry out machine learning, with accessible and reusable tools built of famous libraries. It is entirely open source, and also has a series of user guides and tutorials on different topics, SVMs among others.	https://bit.ly/3szbrAV
Nextflow	The best way to approach Nextflow is to look at the extensive material produced or cataloged by the nf-core community. On their website they list a large number of resources, and also have a series of short “bytesize” webinars covering all the basics.	Tutorials page: https://nfco.re/usage/nextflow Bite-sized webinars (under events): https://nf-co.re/events
Deep Learning	Tensorflow is an open source library for computation, used for DL applications because well equipped to handle multidimensional data arrays (i.e., tensors), and exploit different computing architectures (particularly, GPUs). Its website has a series of very useful tutorials, which we suggest as a starting point to approach this environment.	https://bit.ly/3wbABYo
Deep Learning	If you use R, you will find particularly useful the resource that RStudio has put together on the topic: a large number of tutorials is available for both beginners and advanced R users	https://bit.ly/31sBcXs
Deep Learning	If you prefer a more in depth overview, with conceptual information, Manning Publications offer a video course freely available, which covers a wide range of topics for DL with R	https://bit.ly/3fo1q5H

## Discussion

Biomedical innovation, and in particular research into “omics technologies,” offers the promise of monitoring, preventing and treating age-related disabilities and diseases. Progress in genomics and functional genomics in the past decades have significantly supported our understanding of the molecular mechanisms associated with aging. However, it is nowadays clear that the complexity of aging requires a huge effort into data integration, building a broader omics profile, including genomics, proteomics, lipidomics or metabolomics, transcriptomics, etc. Advances in the comprehension of aging have been made possible thanks to a number of tools and investigative method, like transgenic animal models of aging or epidemiological studies using ‘omics’ tools such as genome wide association and linkage studies.

### Bioinformatics and Omics as a Holistic View on Aging

While the availability of a large amount of data is a clear advantage, there are still many challenges to be solved in order to translate these technological advances into clinical settings. This seems even more challenging in the field of aging, because such an effort requires a more holistic view. Aging is not just the progressive decline of different functions, but rather a well-described phenotype, characterized by a complex remodeling across the whole organism (Franceschi et al., 2000). This is the key reason why omics technologies may greatly improve the definition of different aging phenotypes, and the classification of individuals with features ranging from the very frail, with a poor quality of aging, to the most extreme, the centenarian’s phenotype, characterized by a long life.

### The Power of Diversity

The investment in omics approaches should also represent the opportunity to strengthen diversity in aging research and expanding the wealth of data from underrepresented populations. It is increasingly debated how available data are largely focused on populations of European descent (Peterson et al., 2019). 94.23% of the 488,377-genotyped United Kingdom Biobank participants are of white ancestry (Bycroft et al., 2018); 23andMe dataset has 77% European ancestry (Servick, 2015). Diversity has a serious impact in the quality of the resulting science as well: European ancestry-based polygenic scores derived from GWAS explain only half as much of the variability in the phenotype for non-Hispanic Black ancestry individuals as compared with non-Hispanic White ancestry individuals (Martin et al., 2017). This population heterogeneity may be a reason for the failure to replicate certain findings in other populations (Haiman et al., 2007). Including populations with different ancestry can allow the comparison of data across genetically diverse cohorts, which in turn can provide insights into the underlying pathogenic mechanisms of disease, and a more accurate and population-specific risk assessment. The inclusion of different population backgrounds is particularly crucial in complex traits, like aging, which is determined by a strong geographical component and environmental exposure. A recent study shows that for complex traits, a large proportion of genetic effects are hidden when data across different countries and historical periods are combined (Tropf et al., 2017).

There are many examples of advances facilitated by the inclusion of different populations in the search for age-related traits genetic determinants. For instance, a rare nonsense variant (i.e., which causes the premature termination of a protein) in the gene PCSK9 was found having a higher allele frequency in African Americans: it was associated with a dramatic reduction in low-density lipoprotein cholesterol concentration (LDLC; 28–40%) (Cohen et al., 2005, 2006) and concomitant decrements in coronary heart disease risk (88%) (Cohen et al., 2006). The variant was present in individuals of European descent, but with such a low allele frequency (0.006 vs. 2.6% carriers in African ancestry individuals) to preclude any analysis with sufficient statistical power. It was suggested that the frequency among African ancestry individuals could be a result of selection pressure due to malaria, or to genetic drift. Although identified in the African American population, the study highlighted an important role of PCSK9 variants in molecular mechanisms which play a part in healthy aging: drugs targeting this gene may therefore have a potential benefit for a large number of individuals, beyond the population this role was first identified in.

### The Importance of Community Engagement

Investing in diversity should be achieved also through the increase of meaningful engagement of marginalized communities in the research process. There are wonderful examples of community involvement which could lead an array of initiatives and could be expanded to underrepresented communities. The InCHIANTI study (Ferrucci et al., 2000), mentioned earlier among those selected for re-analysis using new ML methods, 20 years ago adopted an unprecedented level of public engagement: it organized several community events involving study participants, ranging from the promotion of local products characterizing the involved territories, to socializing activities (i.e., knitting and sewing) producing materials used to further promote the social impact of the study. All of this generated reflection and debate around actionable measures, implementing some of the study results.

### The Importance of Sharing

Bioinformatics research thrives when both methods and data can be easily accessed and reviewed in a transparent and open way. For this reason, data sharing becomes crucial also in aging research. There is an increasing receptiveness of the community to this concept now, which we could sum it up with the sentence “open science in open data.” Based on our direct experience, while the most important European research teams in biogerontology collaborated in very large EU-funded research projects, like “European Challenge for Healthy Aging” (ECHA), “Genetics of Healthy Ageing” (GEHA), and “Integrated research on Development determinants of Aging and Longevity” (IDEAL^20^), producing significant progress in the understanding of aging dynamics (De Rango et al., 2008; Beekman et al., 2013; Deelen et al., 2014), data coming from these projects is not available in public repositories or under controlled access repositories. The GEHA project (Skytthe et al., 2011) for example, was launched in 2004 and aimed at sampling an unprecedented number (2500) of non-agenarians sib-pairs from all over the Europe. The project was focused on the analysis of selected chromosomal regions previously associated to the longevity trait, as well as to the discovery of new regions by a whole genome genotyping approach. GEHA represents an example of standard recruitment methodology, both in collecting biological samples and as well as phenotypic information through home-based questionnaires, the latter very crucial for the definition of phenotype (Montesanto et al., 2012). Without recovering this wealth of data into a common data repository, genetic, -omics, and non-genetic data on centenarians, non-agenarians and their families, is kind of lost, and cannot be revisited with the latest bioinformatics methodologies, data integration approaches, nor new data mining methods, which could pave the way to new insights and discoveries in the field, as in the example from Gomez-Cabrero et al. (2021).

Like diversity and inclusion, responsible data and biospecimen sharing was recognized as a scientific imperative by Knoppers (2014), proposing the constitution of The Global Alliance for Genomics and Health (GA4GH), an international, non-profit alliance aimed to accelerate the potential of research and medicine to advance human health and bringing together 600+ leading organizations working in healthcare, research, patient advocacy, life science, and information technology^21^. A similar community should be constituted among the groups working in the field of aging, working together to create frameworks and standards to enable the responsible, voluntary, and secure sharing of genomic and health-related data.

In conclusion, we believe that aging is by definition an omics science. New bioinformatics tools will strengthen this nature and provide new insights into healthy aging, as well as suggest actions to improve our quality of life. We provide here a few recommendations which, in our view, will help and facilitate this development.

### Recommendations

#### Recommendation One: Prioritize Diversity

Researchers should prioritize the inclusion of multiple types of data (ancestral, geographical, environmental, temporal and demographic) and from different populations

##### Benefits

Ancestral diversity strengthens findings, and increases the chance to find actionable mechanisms, thus implementing new strategies to improve quality of life and healthy aging.

#### Recommendation Two: Invest in Data Sharing

Scientists responsible for large population-studies in aging should put an extra-effort in making the data they have collected, especially when funded by public bodies, into accessible repositories.

##### Benefits

The availability of larger datasets, with a wide range of data types, will facilitate reanalysis with new methods, and potentially new insights into the determinants of healthy aging.

#### Recommendation Three: Promote Community-Driven Bioinformatics

Like in other areas of research, there is an increasing need to standardize and share new bioinformatics methods for data mining and omics data integration. This can only be achieved through community discussion and collaborative efforts.

##### Benefits

New pipelines can be developed using artificial intelligence approaches, and they can be available open source to facilitate research activities and analysis of new and existing data.

#### Recommendation Four: Promote Engagement and Participation

Scientists in aging research, and particularly those working on bioinformatics who are often less in contact with the participants of the studies they analyze, should invest in a responsible research and innovation (RRI) for their activities, and dedicate part of their time to community engagement and participation.

##### Benefits

Better awareness about the study impact on quality of life will increase chances for funding, and community engagement will improve both quality of data collection, as well as provide often unexpected insights during the analysis of data.

## Author Contributions

FL conceived the work. FL and SD discussed and selected the content and revised the manuscript. FL, SD, PC, and NR wrote the manuscript. All authors contributed to the article and approved the submitted version.

## Conflict of Interest

The authors declare that the research was conducted in the absence of any commercial or financial relationships that could be construed as a potential conflict of interest.

## Footnotes

1 http://celldesigner.org

2 http://copasi.org

3 http://agefactdb.jenage.de

4 https://www.maelstrom-research.org/mica/network/mindmap

5 http://yu-mbl-muscledb.com/NeuroMuscleDB

6 http://www.metaboage.info

7 https://genomics.senescence.info/index.php

8 https://genomics.senescence.info/genes/

9 https://genomics.senescence.info/longevity/

10 http://libage.ageing-map.org/entries/sources/longevity/

11 https://genomics.senescence.info/species/

12 https://genomics.senescence.info/diet/

13 https://genomics.senescence.info/drugs/

14 https://genomics.senescence.info/cells/

15 http://ageing-map.org/

16 https://www.aginganalytics.com

17 https://www.ncbi.nlm.nih.gov/gap/

18 https://ega-archive.org/

19 https://www.omicsdi.org

20 http://www.ideal-ageing.eu/

21 https://genomicsandhealth.org/

## Supplementary Material

Supplementary Table 1 Available datasets in aging research.
